# Molecular characterization of early breast cancer onset to understand disease phenotypes in African patients

**DOI:** 10.1007/s12032-022-01877-8

**Published:** 2022-11-09

**Authors:** Pamela Derliche Tonouo, Esther Dina Bell, Arnol Auvaker Tiofack Zebaze, Eliane Ndounga, Sidonie Noa Ananga, Etienne Atenguena, Gustave Simo, Abdel Jelil Njouendou, Smiths S. Lueong

**Affiliations:** 1 Faculty of Medicine and Pharmaceutical Sciences, University of Douala, Douala, Cameroon; 2 Department of Medical Oncology Douala General Hospital, Douala, Cameroon; 3 Molecular Parasitology and Entomology Unit (MPEU), Department of Biochemistry, Faculty of Science, University of Dschang, Dschang, Cameroon; 4 Centre Hospitalier Universitaire de Brazzaville, Brazzaville, Congo; 5 Department of Medical Oncology, Yaoundé General Hospital Cameroon and the Cameroon Cancer Registry, Yaoundé, Cameroon; 6 Department of Biomedical Sciences, Faculty of Health Sciences, University of Buea, Buea, Cameroon; 7 Bridge Institute for Experimental Cancer Therapy, West German Cancer Center, University Hospital Essen, Hufeland Str. 55, 45147 Essen, Germany; 8 Division for Solid Tumor Translational Oncology, The German Consortium for Translational Cancer Research (DKTK) and the German Cancer Research Center (DKFZ), Essen/Düsseldorf Partner Site, West German Cancer Center, University Hospital Essen, Essen, Germany

**Keywords:** CAN, TGFß signaling, Early onset of breast cancer, Oncogene, Tumor suppressor

## Abstract

Female breast cancer (BC) is the leading cause of cancer-related deaths worldwide with higher mortality rates and early onset in developing countries. The molecular basis of early disease onset is still elusive. We recruited 472 female breast cancer from two sub-Saharan African countries (Cameroon and Congo) between 2007 and 2018 and collected clinical data from these patients. To investigate the molecular drivers of early disease onset, we analyzed publicly available breast cancer molecular data from the cancer genome atlas (TCGA) and the gene expression omnibus (GEO) for copy number alteration, mutation and gene expression. Early BC onset (EOBRCA) (diagnosis before 45 years) was higher in African women compared with the TCGA cohort (51.7% vs 15.6%). The tumor grade, mitotic index, HER2 + phenotype, basal-like phenotype and ki67 were higher in EOBRCA for all cohorts. BC risk factors such as parity, breastfeeding early onset of menarche and use of hormonal contraceptives were significantly associated with EOBRCA (*p* < 0.05). EOBRCA was equally associated with copy number alterations in several oncogenes including *CDH6* and *FOXM1* and tumor suppressor including *TGM3* and *DMBT1* as well as higher *TP53* mutation rates (OR: 2.93, *p* < 0.01). There was a significant enrichment of TGFß signaling in EOBRCA with *TGM3* deletions, which was associated with high expression of all SMAD transcription factors as well as *WNT* ligands. The Frizzled receptors *FZD1*, *FZD4* and *FZD6* were significantly upregulated in EOBRCA, suggesting activation of non-canonical WNT signaling. Our data, suggest the implication of *TGM3* deletion in early breast cancer onset. Further molecular investigations are warranted in African patients.

**Supplementary Information:**

The online version contains supplementary material available at 10.1007/s12032-022-01877-8.

## Introduction

Breast cancer (BC) has become a leading contributor to the global cancer burden and its incidence largely exceeds the incidence of other malignancies [1], especially in developing countries. Although incidence rates are higher in industrialized countries, mortality rates are higher in developing countries [1]. BC has therefore become a major public health challenge in these countries, especially in sub-Saharan Africa. In most sub-Saharan African countries, poverty, poor health infrastructure, lack of adequate training and lack of awareness constitute a lethal cocktail for BC patients. Unfortunately, very few studies have attempted to understand the molecular underpinnings of female breast cancer traits in these populations. Improving patient welfare in such settings will inevitably require an in-depth knowledge of the clinic-pathological and molecular traits of breast cancer.

Studies in indigenous African (IA) women have revealed increasing incidence with age up to the age of 45 years, after which a decline is observed [2]. Meanwhile, the age-adjusted 5-year overall survival rates are reported to be lower in sub-Saharan Africa compared with North Africa [3]. Although differences in development index might explain the late stage at diagnosis, it is less likely to account for early onset and disease aggressiveness. Additionally, family history of breast cancers has been shown not be associated with early onset of breast cancer [4]. Understanding the molecular traits driving early breast cancer onset and disease aggressiveness is indispensable for precision oncology and preventive measures, especially in low-income countries where prevention is the most achievable combat strategy.

Studies addressing the etiology of EOBRCA have suggested the involvement of toxic environment, disruption of hormonal internal milieu or genetic susceptibility [5]. The underlying genetic susceptibility loci have however remained a mystery. Similarly, studies on occupational, diet and environmental risk factors have led to very little insight. Furthermore, although mutations in high penetrance genes such as *BRCA1* and *BRCA2* are associated with very high risk of contralateral breast cancer, these mutations are only seen in a small fraction of patients with EOBRCA [6, 7]. Very few studies have so far addressed the molecular drivers of EOBRCA both in industrialized and in developing countries. Most importantly, there is little, if at all any information on EOBRCA in indigenous black African population. Comparative analyses of early breast cancer onset in different populations can identify phenotypical similarities that might be driven my identical molecular drivers.

In the light of the aforementioned, we set out to characterize the clinical features of breast cancer in African and western populations and to identify potential molecular traits supporting early onset of breast cancer using publicly available data sets from the TCGA and the gene expression omnibus (GEO). We show that compared with women of other ethnicity, breast cancer development occurs significantly early in African women. Furthermore, BC patients diagnosed before the age of 45 years were more likely to have higher-grade tumors, higher mitotic index and higher rates of Her2 + positivity in both the African and western populations. Molecular analysis of breast cancer data from western populations reveal somatic copy number amplification of several oncogenes including *FOXM1, CDH6, CXCL10* and *NAAA* oncogenes as well as deletions of tumor suppressor genes including *TGM3 DMBT1* and *MYO18B* in patients with breast cancer onset before or at age 45 years. EOBRCA was associated with enrichment in TGFß signaling and epithelial-mesenchymal transition, meanwhile deletion of the tumor suppressor *TGM3* was associated with overexpression of SMAD transcriptions factors, WNT ligands (*WNT5A* and *WNT7B*) as well as the frizzled receptors *FZD1*, *FZD4* and *FZD6* in EOBRCA. These data suggest that tumor suppressor deletion may lead to derepression of TGFß signaling and consequential activation of epithelial-mesenchymal transition to drive early onset of breast cancer.

## Methods

### Patient cohorts

Females with histologically confirmed BC and aged > 18 years were recruited at the Yaoundé general hospital (Cameroon), the Douala General Hospital (Cameroon) and at the University teaching hospital (CHU, Brazzaville) in Congo. Administrative authorizations was obtained from all local sampling sites (N° 1231 DEI-Udo/11/2017/M and N° CBI/395/ERCC/CAMBIN, protocol N° 1086) and all participants gave written informed consent to the study. Participants were prospectively recruited between 2007 and 2018 during routine medical visits and were monitored during the entire study period or until they were disease free, death or opted out of the study. Lifestyle data was collected using a structured internal questionnaire, while clinical phenotypes were retrieved from the individual patient files from each sampling site. Patient data for the TCGA cohort (> 1000 cases) was downloaded from the Genomic data commons (GDC) repository using the TCGAbiolinks Bioconductor package. Additional patient cohort data (> 300 cases) were downloaded from the gene expression omnibus (GSE3494, GSE 5427). All patients with missing age and gender information or who participated for less than six (06) months were excluded from further analysis. Therefore, 115 patients were retained from the Congo cohort, 65 patients in the Yaoundé cohort and the rest were recruited at the Douala general hospital.

### Database mining and in silico analyses

Gene expression data was downloaded from the gene expression omnibus (GEO) for breast cancer patients (GSE3494 & GSE5427) and from the cancer genome atlas (TCGA). Gene expression data was obtained for primary tumors from patients with all stages of BC from the different repositories. Copy number variation data was equally downloaded from the TCGA for the breast cancer project (TCGA-BRCA). All data were downloaded from the GDC legacy (genome assembly hg19) database. For all TCGA data, only data from primary tumors were used, by specifying the “Sample.type” to “Primary tumor”. For copy number variation data, mean copy number segment data from the Affymetrix SNP 6.0 data was used, while illumina Hiseq gene expression quantification data was downloaded for gene expression analysis using the TCGAbiolinks package. Somatic mutation files for TCGA-BRCA were also download as maf files and processes using the maftools Bioconductor package. All non-mutated samples were excluded from the presented oncoplots (but included in the summary statistics). Both gene expression and copy number variation data were analyzed following the TCGAbiolinks user guide. Briefly, copy number segment data was downloaded and filtered on a cut-off threshold of 0.3 for gain or -0.3 for loss. The filtered CNV segment file was then used to create a CNV object. A probe metafile serving as marker matrix was obtained from the Broad institute (ftp://ftp.broadinstitute.org/pub/GISTIC2.0/hg19_support/) and filtered for common CNVs. The filtered marker matrix was the used to create a marker object and reccurent somatic copy number aberrations were identified using the gaia Bioconductor package. CNVs were annotated using the biomaRt and GenomicRanges Bioconductor packages while circus plots were made with the circlize package. TCGA gene expression data was normalized and filtered using the EDAseq package, while edgeR was used for differential gene expression analyses. Genes with a false discovery rate (FDR) of < 0.05 and a log2fold change of at least 1, were considered as differentially expressed. The affy package was used for processing cell files from the GSE 3494 and GSE5427 cohorts. Genes whose expression was affected by CNV changes were obtained by filtering the list of recurrent somatic CNV from the EOBRCA group for all CNVs that were equally present in the LOBRCA. The resulting list was then intersected with the list of differentially expressed genes.

Gene set enrichment was performed using the desktop GSEA application with 1000 permutations on the hallmark gene sets. Only the hallmarks of cancer gene sets were analyzed and gene sets with a FDR < 0.05 and a normalized enrichment score > 1.5 were considered to be significantly enriched. Cluster profiler was used for gene ontology enrichment analysis on differentially expressed genes between EOBRCA and LOBRCA.

### Statistical analyses

The TCGA cohort served as a reference to stratify patients into early and late onsets of BC. Patients were considered to have EOBRCA, if they were first diagnosed before the age of 45 years (mean- 1 SD of the TCGA cohort), or late onset of BC (LOBRCA) for those diagnosed after the age of 50 years. The mean age at diagnosis of BC was determined by computing the column statistics in Graphpad Prism. The survminer package was used to determine the gene expression cut-off for survival analyses, while the latter was performed using survival package. Genes with consistency in CNA pattern and gene expression profiles were used in a multivariate cox proportional hazards model to find associations with patient survival. Relationships between molecular and lifestyle factors and breast cancer phenotypes was assessed by logistic regression. The Kaplan–Meier method was used to compare survival differences and significance was tested using the log-rank test. Several groups were compared using the Kruskal–Wallis test and proportions were compared using the Fisher’s exact test while setting the significance threshold to a *p* value < 0.05. All analyses were performed with the R environment, SPPS or using Graphpad prism software 8.0.0 for Windows, (GraphPad Software, San Diego, California USA).

## Results

**Fig. 1:**
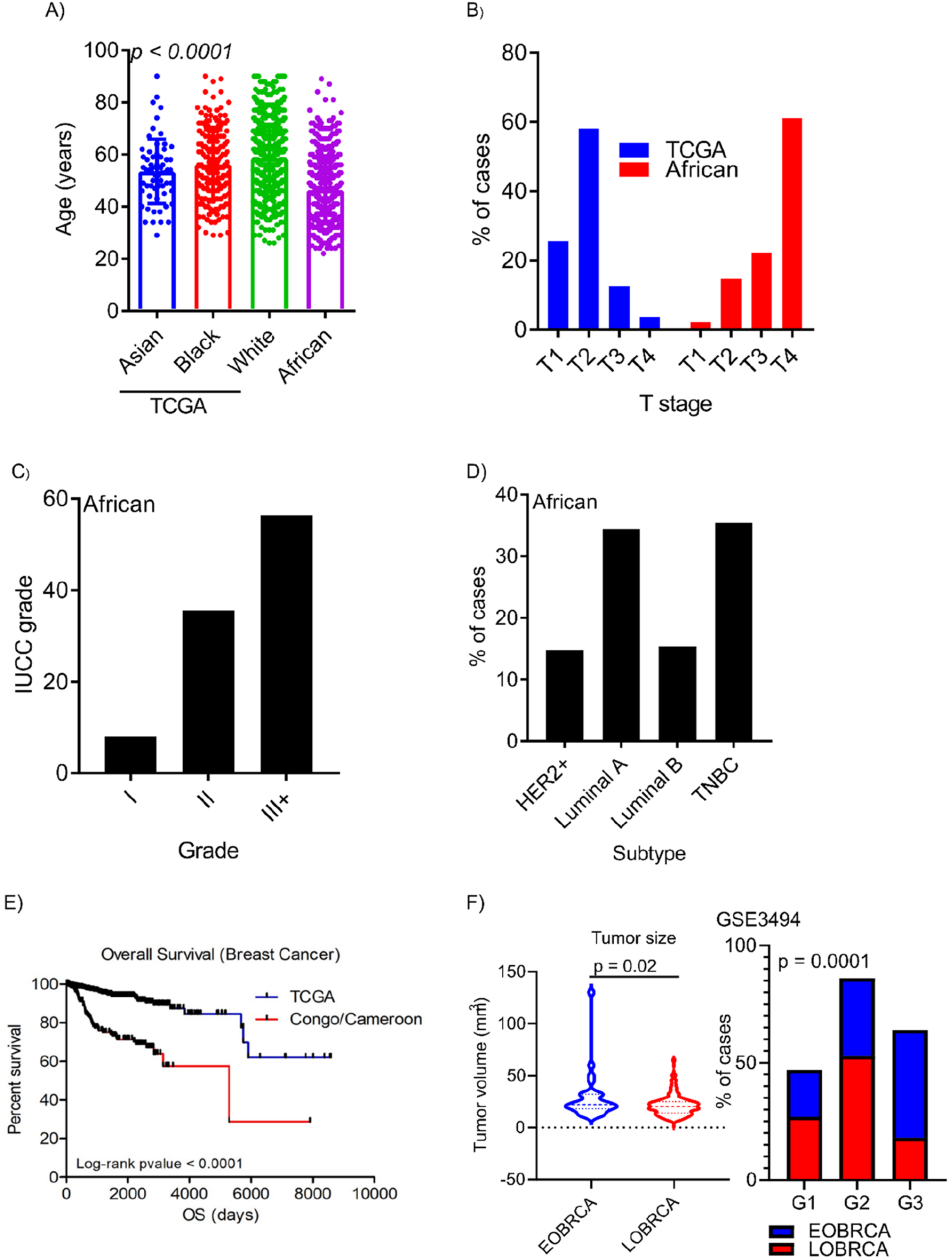
Early onset of highly aggressive breast tumors is prominent among African women. **A** A scatter plot showing the distribution of age at first diagnosis of breast cancer in patients of different ethnicity in the TCGA cohort (*N* = 1018) and in female breast cancer patients from Africa (Cameroon and Congo, *N* = 472). **B** A bar plot showing the distribution of the different tumor stages in the TCGA cohort and in the IA cohort. **C** A bar plot demonstrating the proportions (as percentages) of different tumor grades observed among IA women with breast cancer. **D** A bar plot show the distribution of the different breast cancer molecular subtypes among IA women diagnosed with breast cancer. **E** A Kaplan–Meier overall survival curve representing the survival of breast cancer patients from the TCGA cohort and in SSA women from Cameroon and Congo. Despite the early age at disease onset in SSA women, their overall survival is worse than in the TCGA cohort. **F** A violin plot showing the distribution of breast tumor size in EOBRCA and LOBRCA in among Caucasian women in the GSE3494 study (left panel) and a bar plot showing the distribution of tumor grade in EOBRCA and LOBRCA in Caucasian women (right panel). As observed in SSA women, significantly higher-grade tumors are observed in EOBRCA

We investigated the molecular basis of EOBRCA in African and western populations. The mean age at BC diagnosis was 58.5 ± 13.2 and 46.5 ± 12.9 years for the TCGA and African cohorts, respectively (Fig. 1a). EOBRCA was higher, 244/472 (51.7%) in African patients compared with 159/1018 (15.6%) in the TCGA cohort. Among African women, as shown in Table 1, patients with late disease onset were more likely to have had menarche after the age of 12 years (10% vs 21%, respectively) (OR: 0.41, 95% CI: 0.10–1.42). Furthermore, most of these women had their first pregnancies before the age of 20 years (47.37% vs 58.42%) and most of them did not use contraceptive pills (30.25% vs 41.41%) and breastfed their children for more than 12 months (64.52% vs 43%).

**Table 1:** Patient baseline characteristics

Variables	Sample size 472								P value
	Early onset				Late onset				
	≤ 45 (244) 51.70%				> 45 (228) 48.30%				
Subtype	Luminal A	Luminal B	HER2 Like	TNBC	Luminal A	Luminal B	HER2 Like	TNBC	0.018
	58 (36.25)	25 (15.63)	18 (11.25)	59 (36.87)	61 (33.33)	30 (16.39)	28 (15.30)	64 (34.98)	
Menarche	≤ 12		> 12		≤ 12		> 12		0.0001
	31 (21.53)		113 (78.47)		9 (10)		81 (90)		
Parity	≤ 6		> 6		≤ 6		> 6		0.0001
	117 (80.69)		28 (19.31)		107 (60.80)		69 (39.20)		
Age at first pregnancy	≤ 20		> 20		≤ 20		> 20		0.022
	42 (41.58)		59 (58.42)		60 (52.63)		54 (47.37)		
Contraception	Yes		No		Yes		No		0.0001
	59 (40.41)		87 (59.59)		36 (30.25)		83 (69.75)		
Menopause	Yes		No		Yes		No		0.0001
	12 (7.84)		141 (92.16)		94 (83.93)		18 (16.07)		
Breastfeeding duration	≤ 12 months		> 12 months		≤ 12 months		> 12 months		0.0001
	80 (64.52)		44 (35.48)		36 (43.00)		46 (57.00)		

### Early onset of breast cancer is associated aggressive phenotypes

**Fig. 2:**
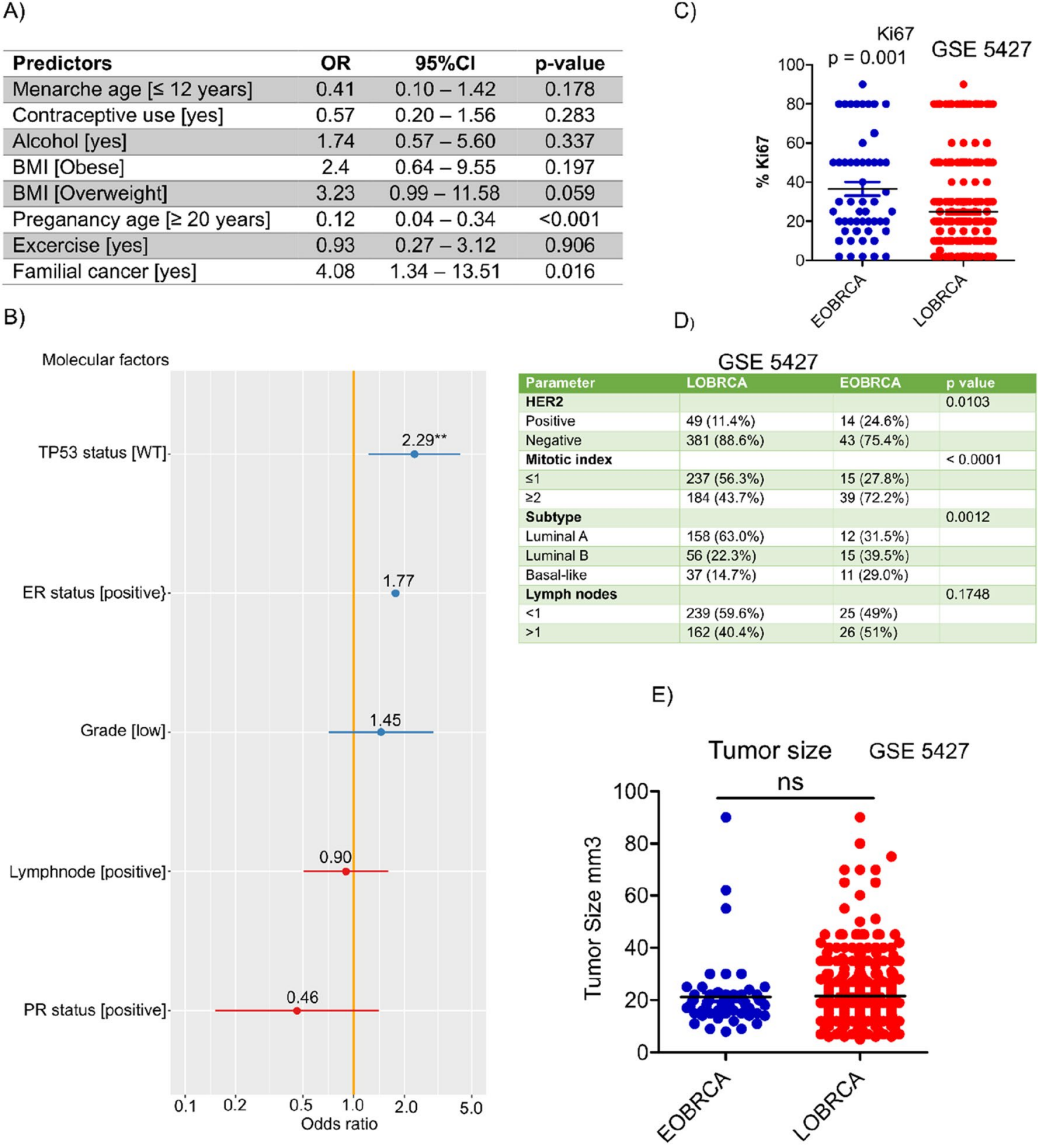
Molecular and lifestyle risk factors are associated with early onset of breast cancer (EOBRCA). **A** A table showing odds ratio for the development of EOBRCA (as reference) from multivariate analyses of lifestyle risk factors collected from IA women with breast cancer. EOBRCA is defined as development of BRCA before the age of 45 years, corresponding to the mean—1SD derived from the TCGA cohort. **B** A forest plot showing molecular factors associated with EOBRCA (as reference). Molecular data was obtained from a publicly available breast cancer cohort (GSE 3494) and patients were stratified to EOBRCA and LOBRCA as described above. **C** A scatter plot showing the percentage of Ki67 expression in EOBRCA and LOBRCA. A significantly higher ki67 is observed in EOBRCA compared with LOBRCA. **D** A contingency table showing the Her2, mitotic index, tumor subtype and lymph node involvement in EOBRCA and LOBRCA. EOBRCA show a significantly higher proportion of Her2 + , higher mitotic index and basal cases compared with LOBRCA. **E** A scatter plot showing the distribution of tumor sizes in EOBRCA and LOBRCA. No significant size difference is observed in tumor size between both groups

Tumor stage analyses revealed, that more than 80% of tumors from the TCGA cohort were T1 and T2 tumors, while about 80% of tumors in African women were T3 and T4 (UICC 7th edition) (Fig. 1b). There was no tumor grade information for the TCGA cohort. In the African cohort however, we observed about 60% of all tumors being grade III (Fig. 1c), meanwhile there was an equal distribution (about 30%) of luminal A and triple negative breast cancer within the African cohort. Her2 + and luminal b tumors accounted for about 15% each (Fig. 1d). Despite the early age at diagnosis of BC in the African cohort, these patients showed significantly poor overall survival compared with the TCGA cohort (Fig. 1e). Similar to the African cohort, significantly higher tumor grades were observed in patients diagnosed with BC before the age of 45 years in other GEO cohorts (Fig. 1f). To identify lifestyle and molecular features associated with early onset of BC, we performed a multivariate logistic regression on data from the African cohort. Early onset of menarche (≤ 12 years as well as the use of contraceptive pills were associated with higher odds of developing BC before the age of 45 years. A significant association was also seen between EOBRCA and late age at first full pregnancy, (OR: 0.12, 95% CI: 0.04–0.34, *p* value < 0.001) (first pregnancy at age ≥ 20 years). Family history of breast cancer was significantly associated with LOBRCA (OR: 4.08, 95% CI: 1.34–13.51, *p* = 0.016). (Fig. 2A). Using molecular data from publicly available Breast cancer studies (GSE3494), we analyzed the relationship between different molecular features and early onset of BC. Of all features analyzed, only *TP53* mutational status was significantly associated with age-dependent breast cancer development. Wild type *TP53* status was significantly associated with late onset of breast cancer (Fig. 2b). We further analyzed another BC cohort (GSE5427) with ki67, mitotic index, HER2 status, tumor subtype and lymph node involvement. As seen in Fig. 2c, significantly higher ki67 positivity rates were observed in EOBRCA. Additionally, higher rates of Her2 positivity, higher mitotic index, and higher proportions of basal-like tumors were associated with EOBRCA. (Fig. 2d).There was no significant difference in the tumor size between EOBC and LOBRCA (Fig. 2e).

### Higher rates of *TP53* mutations in EOBRCA

**Fig. 3:**
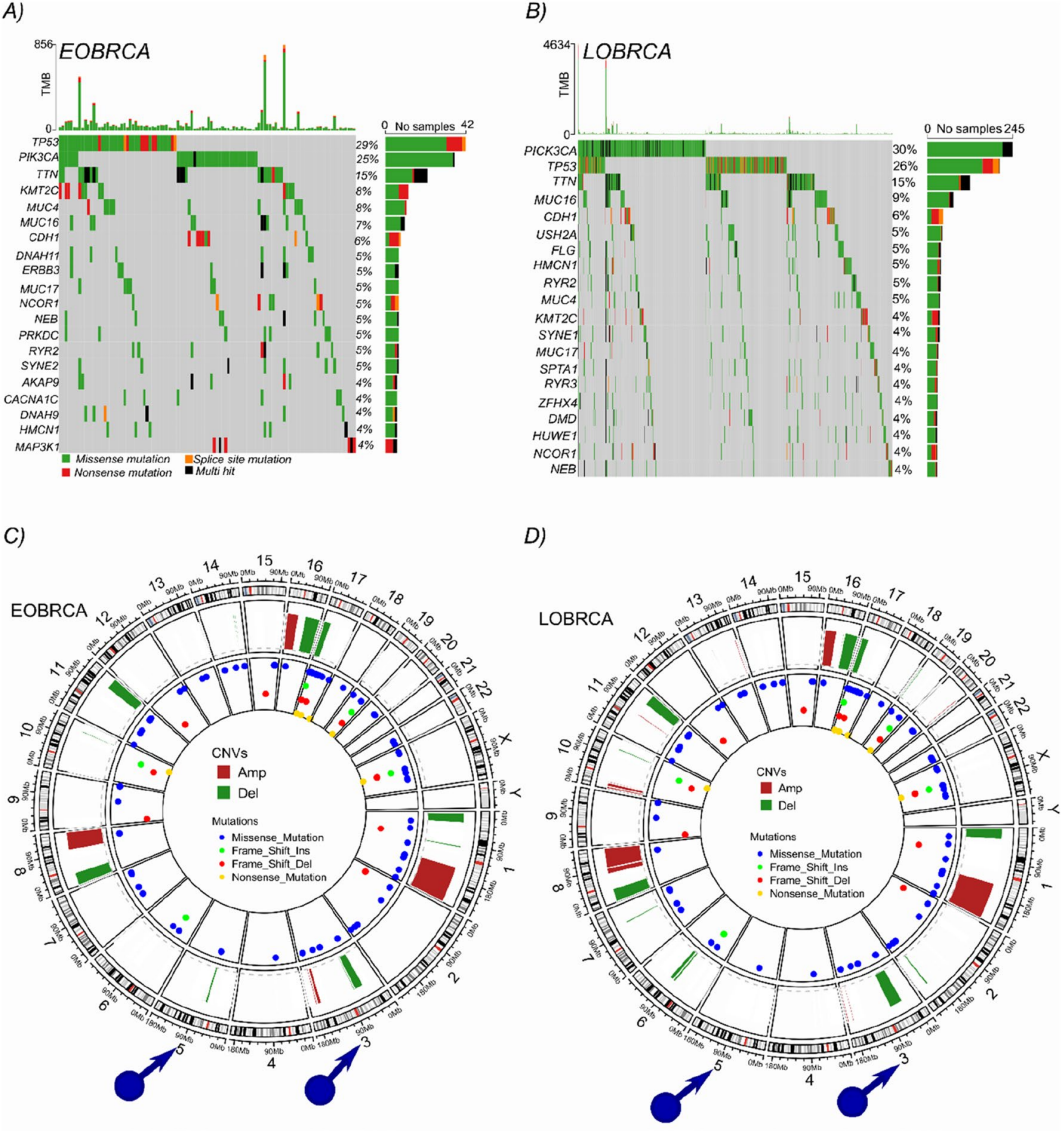
Genetic characterization of EOBRCA reveals specific patterns in PIK3CA and *TP53* mutations and differences in somatic copy number alterations. **A** An oncoprint showing the top 20 most mutated genes in EOBRCA breast cancer patients from the TCGA cohort (samples without any mutation are excluded from the plot but included in the statistics). **B** An oncoprint showing the top 20 most mutated genes in LOBRCA breast cancer patients from the TCGA cohort (samples without any mutation are excluded from the plot but included in the statistics). **C** A circus plot, showing a summary of all somatic copy number alterations (green = deletion and red = amplifications) and somatic mutations in the different chromosomes in patients with EOBRCA from the TCGA cohort. **D** A circus plot, showing a summary of all somatic copy number alterations (green = deletion and red = amplifications) and somatic mutations in the different chromosomes in patients with LOBRCA from the TCGA cohort. For computational reasons, the first 200 patients in the LOBRCA ware analyzed, leading to comparable sizes in the EOBRCA (159 cases) and LOBRCA (200 cases)

After observing associations between *TP53* mutations and EOBRCA, we analyzed somatic mutations in breast cancer data from the TCGA. The analyses revealed that both *TP53* and *PIK3CA* were the most predominantly mutated genes in BC. However, a higher rate of *TP53* mutations were observed in patients with EOBRCA, compared with LOBRCA patients (29% vs 26%, respectively Fig. 3a). on the other hand, *PIK3CA* mutations were predominant in LOBRCA compared with EOBRCA (30% vs 25%, respectively Fig. 3b). Given that these mutations accounted for less than 50% of all EOBRCA cases, we further analyzed copy number alterations in both patient cohorts. As shown in the circus plots in Fig. 3c, d, although EOBRCA and LOBRCA showed similar CNA patterns, there were more CNA events in LOBRCA compared with EOBRCA. Interestingly, remarkable differences were observed in certain chromosomes, especially chromosomes 3, 5 and 14 in EOBRCA. In this group, there was a remarkable genomic amplification, which was almost absent in LOBRCA. Similarly, on chromosomes 5 and 14 in EOBRCA, there was an obvious deletion, which was not observable in LOBRCA.

### EOBRCA is associated with CNA in oncogenes and tumor suppressors

**Fig. 4:**
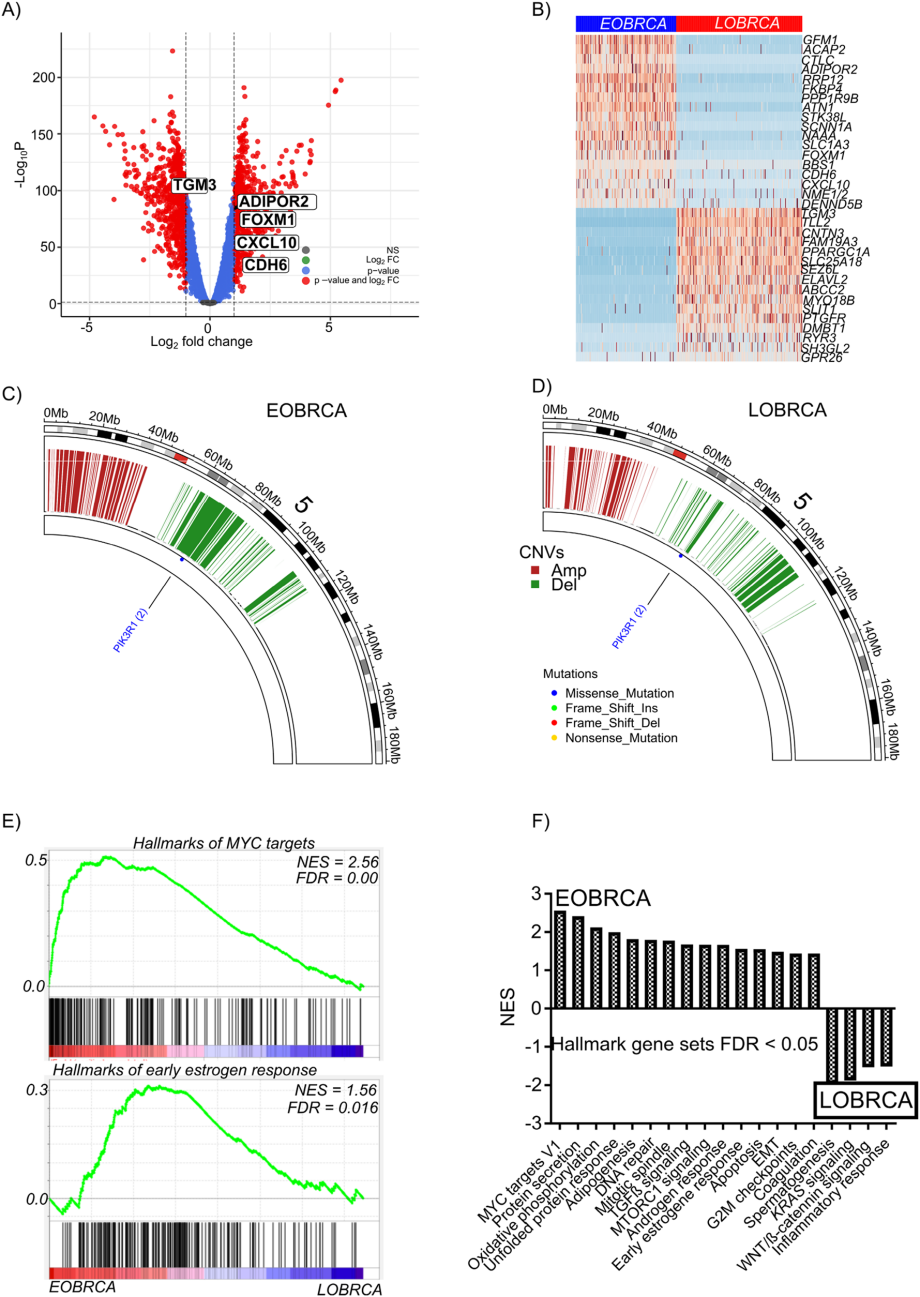
Somatic copy number alterations affect the expression of tumor suppressor and oncogenes in EOBRCA. **A** A volcano plot, showing differentially expressed genes between EOBRCA and LOBRCA in patients from the TCGA cohort. Key amplified oncogenes and deleted tumor suppressor genes are marked. **B** A heatmap showing differentially expressed genes with somatic copy number amplifications or deletions in EOBRCA. **C** A representative partial circus plot of chromosome 5 showing a zoom out of the copy number alterations and mutations in EOBRCA. **D** A representative partial circus plot of chromosome 5 showing a zoom out of the copy number alterations and mutations in LOBRCA. **E** Gene set enrichment plot showing representative hall mark gene sets enriched in EOBRCA compared with LOBRCA in the TCGA cohort. **F** A bar plot showing all enriched hallmark gene sets with false discovery rate (FDR) < 0.05 in EOBRCA and LOBRCA

We investigated if the observed CNA differences between early and late disease onset affected gene expression and cancer development. We specifically focused on genes with CNA exclusively in EOBRCA. To this end, we filtered out all genes with CNA in both EOBRCA and LOBRCA and generated a list of EOBRCA CNAs (supplementary Table 1). We performed differential gene expression analysis for all patients in both groups and selected all genes with a FDR < 0.05 and an absolute log2 fold change greater than 1, to constitute a list of differentially expressed genes (Fig. 4a, and supplementary Table 2). Our list of differentially expressed genes was then intersected with the EOBRCA CNA list and resulted in 61 genes (supplementary Table 3). From this table, 18 genes showed similar trends in CNA type (amplification & deletion, supplementary Table 4) and gene expression patter (upregulation and down regulation). Of these 34 genes, 17 genes with copy number amplifications were upregulated, while 17 genes with copy number deletions were down regulated (Fig. 4b). Of note, several cancer associated genes such as *CDH6, FOXM1, NAAA* and *CXCL10* [8–12] were amplified and upregulated. Candidate tumor suppressor genes such as *TGM3, MYO18B, SH3GL2, DMBT1, SEZ6L* and *SLIT1* were equally deleted [13–20]. Further investigation in CNA events in EOBRCA and LOBRCA, indeed indicated, that between 30 and 40 Mb on chromosome 5, in the region where *CDH6* is located, there was a strong copy number amplification in EOBRCA (Fig. 4c), compared with LOBRCA (Fig. 4d). There was equally a very pronounced genetic deletion between 60 and 100 Mb on chromosome 5 of EOBRCA, which was barely seen in LOBRCA (Figs. 4c, d). These observations, indeed confirmed, that the observed upregulation of *CDH6* in EOBRCA was not epigenetically regulated but associated with gene amplification.

In order to understand the hallmarks of EOBRCA, gene set enrichment analysis was performed on gene expression data of both groups. Hallmarks of *MYC* targets as well as early estrogen response were highly enriched in EOBRCA (Fig. 4e). Other well know hallmarks of cancer such as TGFß and mTORC1 signaling were equally highly enriched in EOBRCA. In LOBRCA, *KRAS*, WNT/ß-catenin and hallmarks of inflammatory response were enriched (Fig. 4f). Comparing the enriched hallmark gene sets in both groups, we observed that there were more cancer hallmark gene sets enriched in the EOBRCA compared with only 4 genes sets (with FDR < 0.05) enriched in LOBRCA. Most importantly, gene sets associated with highly aggressive tumors such as the hallmarks of *MYC* targets, *TGFß* as well as mTORC1 were strongly enriched in EOBRCA [21, 22].

### Altered genes are associated with stemness and tumor suppression

Gene expression analyses revealed that some amplified genes were upregulated in EOBRCA, while some deleted genes were downregulated. We then asked if the amplified genes had any tumorigenic properties that may explain their role in early disease onset. As seen in Supplementary Fig. 1, the cancer hallmark gene, *FOXM1* was upregulated in almost all EOBRCA and not in LOBRCA. *FOXM1* is a master transcription factor, regulating tumor cell proliferation, self-renewal and tumorigenesis in several human cancers [23]. Similarly, *CDH6*, another EMT-promoting gene was upregulated in most of the EOBRCA cases. Furthermore, another amplified gene, *PPP1R9B*, has been associated with tumor progression and stemness [24] in human tumors. Among the deleted and downregulated genes, several of these genes were previously reported to have tumor suppressor properties in some solid tumors. In lung cancer for example, the deleted gene *MYO18B* was reported as a tumor suppressor [19]. Another deleted gene, *TGM3*, is also know to play tumor suppressor roles in colorectal cancer by repressing EMT and PIK3/AKT signaling [25]. In urothelial carcinoma, deletion of the gene *SH3GL2* is known to promote malignant behavior [17], meanwhile while *DMBT1* has been proposed as a tumor suppressor in brain cancers [18].

### EOBRCA CNA gene expression patterns is prognostic

**Fig. 5:**
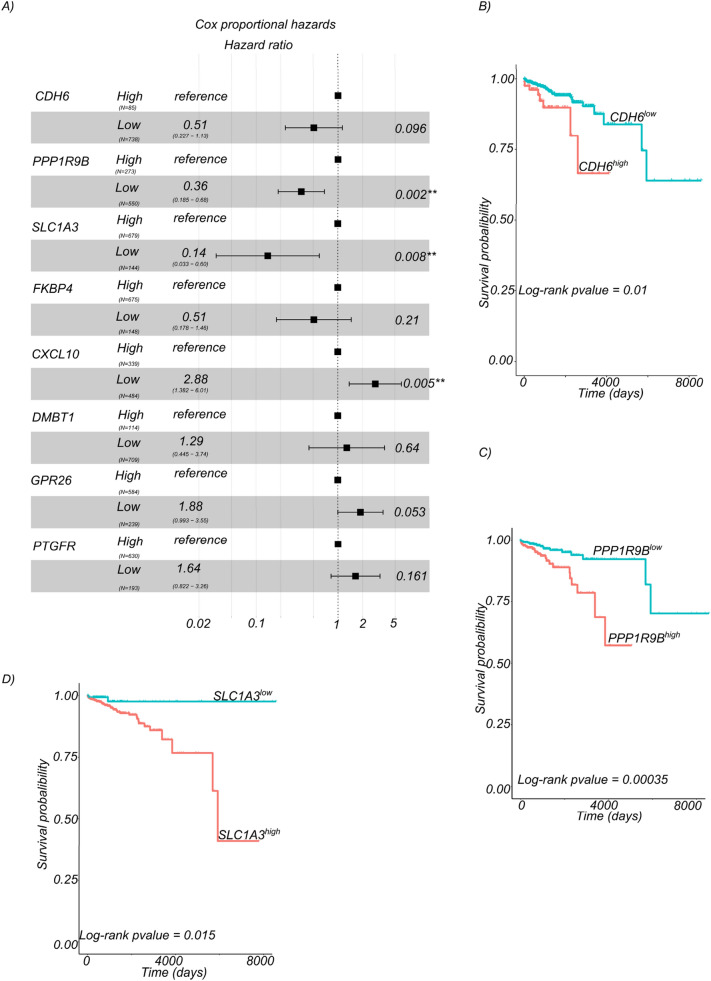
The expression of oncogenes and tumor suppressors with somatic copy number alterations in EOBRCA have prognostic value in BRCA. **A** A forest plot showing multivariate cox proportional hazards ratios for the association of genes with somatic copy number alteration and breast cancer patient survival. Data is shown for all patients with LOBRCA from the TCGA. **B** Kaplan–Meier overall survival curve for patients with LOBRCA from the TCGA cohort. Patients were stratified for the expression of the EOBRCA amplified gene *CDH6*. The stratification cut-off was statistically determined using the survminer package. **C** Kaplan–Meier overall survival curve for patients with LOBRCA from the TCGA cohort. Patients were stratified for the expression of the EOBRCA deleted gene *KLRB1*. The stratification cut-off was statistically determined using the survminer package

We then investigated possible associations between patient survival and somatic copy number alterations. As shown in Fig. 5a, in a multivariate cox regression, low expression of *CDH6* was significantly associated with better survival, (HR: 0.51, 95% CI: 0.23–1.13, *p* = 0.096). Similarly, low expression of *PPP1R9B* and *SLC1A3* were associated with better patient survival (HR: 0.36, 95% CI: 0.18–0.68, *p* value = 0.002 & 0.14, 95% CI: 0.033–0.6, p value = 0.008, respectively). Low expression of *CXCL10* was contrarily significantly associated with poor survival (HR: 2.88, 95% CI: 1.38–6.1, *p* value = 0.005) meanwhile low expression of the deleted tumor suppressor genes *DMBT1* was not significantly associated with poor survival (HR: 1.29, 95% CI: 0.44–3.74, *p* = 0.64). Low expression of *GPR26*, another EOBRCA deleted gene was associated with poor survival. The expression of other EOBRCA deleted and downregulated genes were not significantly associated with survival. Kaplan–Meier survival analysis revealed, that patients with low expression of *CDH6* lived significantly longer than patients with higher expression log-rank *p* = 0.01, (Fig. 5b). Similarly, patients with low expression of *PPP1R9B* lived significantly longer than those with higher expression log-rank *p* = 0.00035, (Fig. 5c), while low expression of *SLC1A3* was also associated with better overall survival, log-rank *p* = 0.015 (Fig. 5d).

### TGFß signaling is activated *TGM3*^*low*^ EOBRCA

**Fig. 6:**
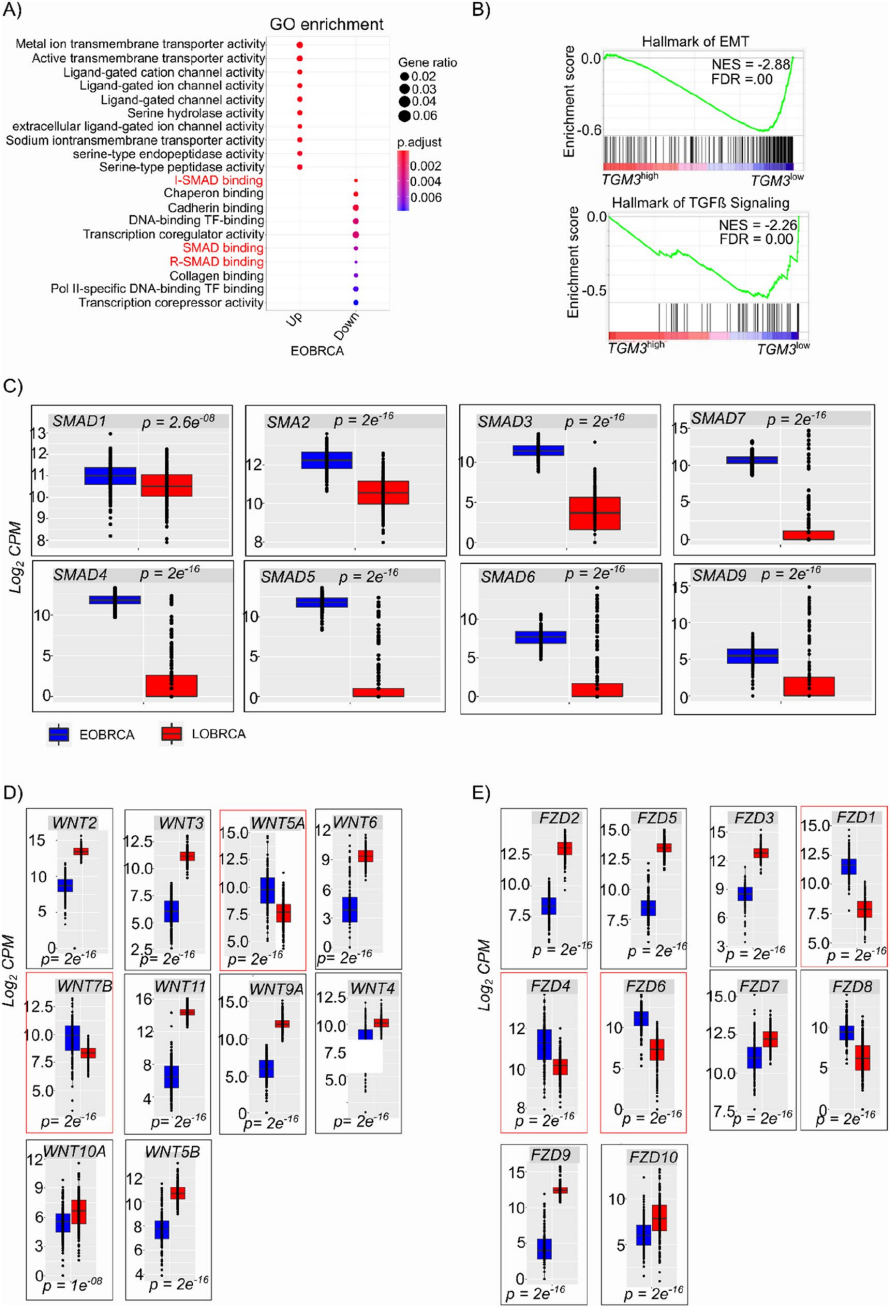
Deletion of TGM3 is associated with activation of TGFß and epithelial to mesenchymal signaling. **A** Gene ontology enrichment dot plot for genes differentially upregulated and downregulated in EOBRCA. A strong enrichment in SMAD binding is seen in EOBRCA. **B** Gene set enrichment plot showing enrichment in the hallmarks of epithelial to mesenchymal transition as well as enrichment in TGFß signaling in*TGM3*^*low*^ breast cancer samples. **C** Boxplots showing the expression of all SMAD genes in EOBRCA and BRCA genes. Statistically significant higher expression of almost all SMAD genes is observed in EOBRCA. **D** Boxplots showing the expression of all WNT genes in EOBRCA and LOBRCA genes. Statistically significant higher expression of almost non-canonical WNT ligands (*WNT5A* & *WNT7B*) genes in EOBRCA. **E** Boxplots showing the expression of all FZD genes in EOBRCA and LOBRCA. Statistically significant higher expression three FZD genes is observed in EOBRCA. The non-canonical WNT receptors FZD6 is significantly higher in EOBRCA compared with LOBRCA

To further understand pathways driving EOBRCA, we performed gene ontology analysis on gene that were differentially expressed in EOBRCA using cluster profiler. Significant downregulated genes were strongly enriched for SMAD binding and transcription factor activity (Fig. 6a). We then investigated if low expression of the tumor suppressor *TGM3* is associated with similar pathways. As seen in Fig. 6b, gene set enrichment analyses revealed an enrichment in the hallmarks of TGFß signaling and epithelial-mesenchymal transition in samples with low expression of TGM3. Interestingly, almost all SMAD genes were highly expressed in EOBRCA (Fig. 6c). Further analyses revealed that the non-canonical Wnt ligands WNT5A and WNT7B were the only significantly upregulated ligands in EOBRCA (Fig. 6d). Lastly, the Frizzled receptor *FZD6*, but also *FZD1* and *FZD4* were upregulated in EOBRCA (Fig. 6e). It is very likely, that early onset of breast cancer is associated with non-canonical Wnt signaling. This might result for the derepression of the WNT/ß-catenin signaling pathway by downregulation of the tumor suppressor *TGM3* in cases of EOBRCA.

## Discussion

We investigated EOBRCA in African and Western populations and observed more than 50% of early disease onset in the African cohort, compared with the only about 15% in western populations. Analysis of BC risk factors revealed that family history of BC was not related with early onset of BC, as postulated in previous findings [4]. More than 60% of African patients had stage IV tumors, which might be explained by lack of awareness and limited resources coupled with customs and traditions. Higher ki67, higher tumor grades, high mitotic index and more basal-like phenotypes were observed in EOBRCA irrespective of ancestry. Interestingly, meanwhile the aggressive TNBC subtype represents a minor subtype in other populations (10–15%) [26], we observed about twofold increase rates in TNBC among African women. EOBRCA among African women was significantly associated with several well known risk factors such as lower breastfeeding duration, higher rates of contraceptive use, late age at childbirth and early onset of menarche [27]. Molecular analyses revealed higher rates of *TP53* mutation in BC patients with early disease onset. In effect, higher rates of *TP53* mutations have been reported in highly aggressive breast tumors [28, 29]. *TP53* loss of function mutations compromises DNA damage repair and cell cycle control that may drive early cancer onset. Analysis of hallmarks of cancer in patients with early disease onset within the TCGA cases, revealed the enrichment of gene sets associated with tumor aggressiveness such as the hallmarks of *MYC* targets, mTOCRC1 and TGFß-signaling. In effect, higher *MYC* expression has been reported in aggressive breast tumors and is a driver of epithelial-mesenchymal transition [30, 31]. Oncogene amplifications and inactivation of tumors suppressors are hallmarks of cancer development [32, 33]. Somatic amplification of key oncogenes such as *FOXM1* and *CDH6* where characteristic of EOBRCA. Similarly, several tumor suppressor genes such as *TGM3* and *DMBT1* were equally deleted in EOBRCA. The transcription factor *FOXM1* is an established master regulator of tumorigenesis across several human cancers [34] and was exclusively amplified in patients with EOBRCA. Similarly, the oncogene *CDH6* is a well-known oncogene and has been linked with poor outcome in other cancer entities [9]. *CDH6* is also known to promote EMT and metastasis in cancer [8]. *CDH6* is also responsible for cellular adhesion and invasion in renal and ovarian cancers [35]. Additionally, *DMBT1,* a tumor suppressor involved in immune defense and epithelial differentiation was deleted and downregulated in EOBRCA. In effect, this gene was previously shown to be down regulated in BC, although the underlying mechanism remained unclear [36]. We now show, that downregulation of this gene is predominantly in EOBRCA and is mediated by copy number deletion. *TGM3,* a gene that functions as a tumor suppressor and in repressed in several cancer entities [13, 25] was also deleted and downregulated in EOBRCA. In effect, *TGM3* has been proposed to be a tumor suppressor by repressing EMT and PIK3/AKT pathway in colorectal cancer [25]. Downregulation of *TGM3* in EOBRCA was associated with epithelial-mesenchymal transition as well as TFGß signaling. Almost all SMAD genes were upregulated in EOBRCA, meanwhile only the non-canonical Wnt ligands (*WNT5A* and *WNT7B*) were upregulated in EOBRCA. Similarly, the non-canonical receptors *FZD6*, but also FZD4 and *FZD1* were upregulated in EOBRCA. Deletion of *TGM3* might therefor lead to derepression of non-canonical Wnt signaling, thereby activating epithelial-mesenchymal transition to drive early disease onset and aggressiveness. Other candidate tumor suppressor genes gene such as *SLIT1*, *SEZ6L and MYO18B,* were deleted in EOBRCA and consequently downregulated at the gene expression level, as reported in other solid tumors [16, 20, 37]. The deletions of these genes in patients with early onset of BC might indeed render patients more susceptible to cancer development upon exposure to other BC risk factors. Meanwhile much still needs to be done to understand the molecular underpinnings of early breast cancer onset in African women, the present study provides a firm background for potential areas that might be exploited and reveals genomic alterations that might be explored for the development of biomarkers for early detection of BC. Furthermore, our data has revealed the possible implication of age-associated molecular differences in treatment outcome and open new horizons that might help in fine-tuning the design of clinical trials.

## Supplementary Information

Below is the link to the electronic supplementary material.

Supplementary file1 (DOCX 209 kb)

Supplementary file2 (XLSX 75 kb)

Supplementary file3 (XLSX 137 kb)

Supplementary file4 (XLSX 16 kb)

Supplementary file5 (XLSX 52 kb)
