# Supplementary material for: Molecular characterization of early breast cancer onset to understand disease phenotypes in African patients
Source: Med Oncol. 2022 Nov 9;40(1):13. doi: 10.1007/s12032-022-01877-8 (PMC9646617; doi:10.1007/s12032-022-01877-8)
Supplement: Supplementary file 1 — Supplementary file1 (DOCX 209 kb) [file 12032_2022_1877_MOESM1_ESM.docx]

**Supplementary figure 1**: Copy number alterations in key oncogenes and tumor suppressor genes alter their expression in EOBRCA and LOBRCA. Boxplots, showing the expression of the amplified and upregulated oncogenes *FOXM1, CDH6, CXCL10* and *PPP1R9B* as well as deleted and downregulated candidate tumor suppressor genes *TGM3, SEZ6L, SLIT1* and *DMBT1* in EOBRCA and LOBRCA.

**
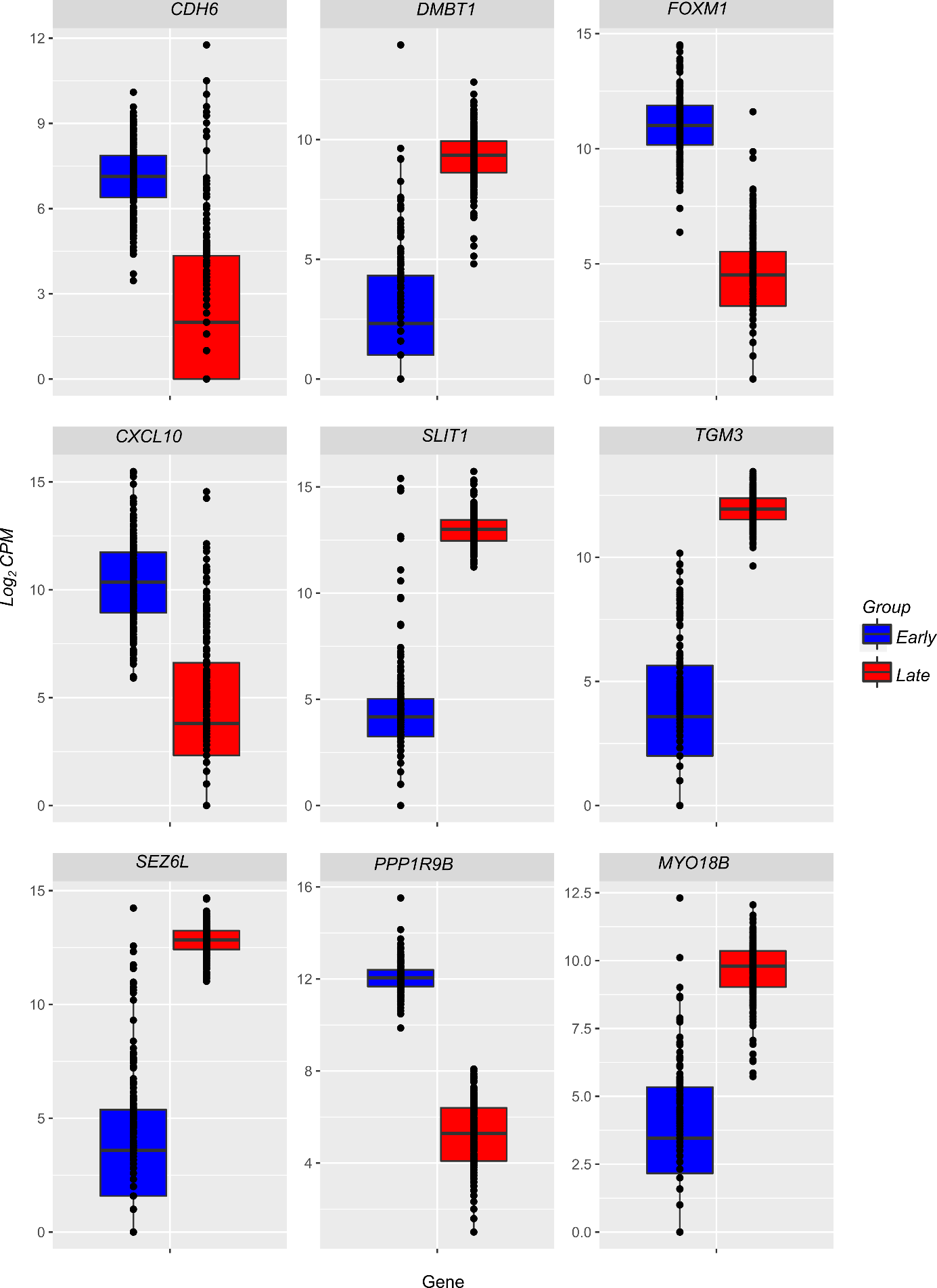
**
